# All-cancer incidence and mortality in Pakistanis, Bangladeshis, and their descendants in England and Wales

**DOI:** 10.1186/s12889-024-20813-1

**Published:** 2024-12-02

**Authors:** Joseph Harrison, Frank Sullivan, Katherine Keenan, Hill Kulu

**Affiliations:** 1 School of Geography and Sustainable Development, University of St Andrews, Irvine Building, North Street, St Andrews, KY16 9AL UK; 2 School of Medicine, University of St Andrews, St Andrews, UK

**Keywords:** Migration, Health, Cancer, Immigrant mortality, Survival analysis, England and Wales, Immigrant cancer

## Abstract

**Background:**

This paper identifies differences in all-cancer incidence and mortality between Pakistani-born (PB), Bangladeshi-born (BB), their descendants, and the White British (WB) in England and Wales. Pakistanis and Bangladeshis are the most marginalised and disadvantaged groups in England and Wales yet, are found to have low cancer mortality and low all-cause mortality. Previous studies though have not looked at generational differences, applied individual-level data nor separated Pakistanis and Bangladeshis from each other and other Asian groups.

**Methods:**

We use the Office for National Statistics Longitudinal Study of England and Wales which is a 1% representative sample of the population. We apply event history analysis on a study period from 1971 to 2016, following individuals from age 20 until a first cancer incidence, censoring at emigration or death. We observe 10,885,500 person-years and 71,926 cancer incidences for WB; 125,700 person-years and 295 events for PB; 53,900 person-years and 113 events for BB and 26,900 person-years and 24 events for descendants. Following incidence, we study a maximum of ten years until a death from cancer, or censoring. In this second analysis on mortality our sample has 329,700 person-years and 31,689 cancer deaths for WB; 1,200 person-years and 104 events for PB; 400 person-years and 50 events for BB and 100 person-years and 10 events for descendants.

**Results:**

Results from the fully adjusted models show that the risk of cancer incidence is lower for PB, BB and descendants compared to the WB native group. Estimated hazard ratio (HR) equals 0.42 for PB (95% confidence interval (CI): 0.38–0.47), for BB HR is 0.38 (CI: 0.32–0.46) and, for descendants HR is 0.36 (CI: 0.24–0.54). Results for cancer mortality after incidence show HR for PB is 0.93 (CI: 0.76–1.12), for BB it is 0.95 (CI: 0.72–1.25) and for descendants HR equals 1.62 (CI: 0.87–3.02 - significant at 90%).

**Conclusions:**

Using high quality representative data, we show that lower incidence of cancer and not better survival is the driver of the low cancer mortality previously found. This advantage persists across immigrant generations, but all-cancer mortality following incidence may be elevated for descendants.

**Supplementary Information:**

The online version contains supplementary material available at 10.1186/s12889-024-20813-1.

## Background

The stock of immigrants in the United Kingdom (UK) is substantial, approximately 15% are foreign-born, former British colonies such as Pakistan and Bangladesh contribute large numbers of these, over half a million born in Pakistan and over a quarter of a million in Bangladesh [1]. This long-term settlement now means there are many UK-born descendants of immigrants from both Pakistan and Bangladesh. Cancer is one of the most common causes of mortality in high-income countries. In the UK 27% of deaths in 2021 were due to neoplasms, a level which has been consistent over previous decades and is similar to other high-income countries [2]. Therefore, understanding the differences in cancer incidence and mortality between immigrants and majority populations has long-term health policy implications [3].

This study investigates cancer incidence and mortality in Pakistani and Bangladeshi immigrants, and their descendants in England and Wales. We focus on these groups as they represent the most marginalised minority groups in the UK. Previous studies have combined Pakistanis and Bangladeshis together (often with Indians too) [4–6]. Whilst there are similarities in the timeline of migration from Pakistan and Bangladesh and prior to 1971 they were one nation, it is important to recognise that these countries are separated by India. This geographic difference could mean that early life conditions immigrants were subjected to have differed. Further, these regions comprise distinct cultures, ethnic, and linguistic groups. These distinctions mean migration experiences and selection has differed which has materialised in different (albeit similar) residential and socioeconomic outcomes [7, 8]. Moreover, the boundaries of ethnic groups are symbolic of different practices of, language, religion, and culture. Combining groups can undermine these differences and the specific racialised experiences of immigrants and their descendants, thus research should reflect the richness of diversity in destination contexts and where possible maintain these distinctions [9].

In addition to disaggregating Pakistani and Bangladeshi immigrants, this study makes further contributions. We investigate differences in both the risk of cancer incidence and risk of subsequent mortality; previously studies have looked at either incidence [4, 5, 10] or mortality [6, 11–14]. Those which have looked at both focus on specific cancers in specific geographic areas of England [15–17]. Moreover, we take a generational approach and compare immigrants to descendants, something which very few studies in the England and Wales context have done [13].

### Cancer incidence and mortality in immigrants

Selection into migration and the higher likelihood of positive health behaviours are two of the key factors which contribute to lower mortality in immigrants compared to natives [18–21]. Those who move from low-income to high-income settings tend to have a larger advantage. Upon migration chronic morbidities linked to ‘western’ lifestyles are uncommon, whereas risks from infection-related diseases are higher. The better healthcare infrastructure in the destination immediately reduces the risk of mortality from infectious diseases and is known as the ‘rapid health transition’ [22, 23]. The advantage reduces the longer the duration of stay, due to the development of more negative health behaviours by immigrants [19, 20, 22]. A ‘double burden’ can also exist, where early life exposures in the origin continue to be a health risk at older ages alongside negative health behaviours acquired in the destination [23].

Immigrant cancer studies have been conducted in various contexts (see [24] for a review). Research using population registers in Sweden [25–28], Belgium [29, 30], the Netherlands [31], and Norway [32, 33] have consistently found lower cancer incidence and mortality for immigrants, particularly those from low-income countries.

Cancers linked to early life infections such as liver or stomach are more common in immigrants from low-income countries, whereas those from high-income origins and native populations are more susceptible to lifestyle driven cancers such as lung and breast cancer [24]. Risks from cancers which are caused by microbial infections and nutritional imbalances remain high for some immigrants no matter the duration in the destination [34]. These findings suggest that the pattern of genetic cancer risk may be set in early years in the origin country [34].

### Cancer incidence, and mortality among the descendants of immigrants

Studies on cancer incidence and mortality in descendants are less common due to their younger age, which means less statistical power and fewer observable cancer events. However, the risk of cancer is believed to approximate to native levels within one or two generations [3]. The aetiologies of cancer in descendants differs from that of their parents. There may be some genetic inheritance of susceptibility or protection from immigrants parents [22]. However, ‘unhealthy assimilation’ and increasing negative health behaviours associated with high-income countries means lifestyle driven cancers increase compared to the immigrant generation [29, 35].

European studies have found that second-generation immigrants whose background is a similar high-income country have cancer incidence and mortality that is more comparable to natives compared to their parents [29, 36]. When the parental origin is a low-income country there is variation, second-generation Moroccans in the Netherlands had lower all-cancer mortality risk compared to native Dutch, but no other second-generation group have the same advantage [31]. Results from the United States also show differences in second-generation cancer risks by gender [37, 38].

### Cancer in Pakistanis and Bangladeshis in England and Wales

All-cause mortality in England and Wales is lower for those born in South Asia, with low cancer mortality a contributing factor to this [12]. Prior studies on immigrant and minority cancer in the UK context tend to either use ethnicity, thus combing immigrants with their descendants [4, 5, 15, 16], or use only country of birth therefore only studying immigrants [10–12, 14]. For studies which use ethnicity, findings show that the broad ethnic group of Asians (which includes Chinese) has lower all-cancer incidence, compared to White British, this holds across most sites, with exceptions being gallbladder, Hodgkin lymphoma, liver, and thyroid cancers [4]. The broad South Asian ethnic group also has better survival after cancer onset, although this has narrowed in recent years [6]. Evidence from site-specific studies indicate that ethnic Pakistanis and Bangladeshis have lower incidence of breast and prostate cancer compared to equivalent White British population, but similar chance of survival after diagnosis [15, 16]. For liver cancers, ethnic Pakistanis and Bangladeshis have higher incidence than the majority [17]. Lifestyle driven cancers have been increasing in South Asians over time [5], illustrating that acculturation to negative health behaviours does occur, corroborating findings from other European contexts [24].

For studies which use country of birth, all-cancer mortality was lower for Pakistani-born men and women but has shown convergence with native levels over time [14]. Mortality from lung, colorectal, breast, and prostate cancer are all lower for the Pakistani and Bangladeshi-born population compared to natives- except lung cancer in Bangladeshi men [11]. Another study found that all-cancer mortality was lower for Pakistani and Bangladeshi immigrants, site-specific analysis showed lower or non-different risks for all sites except liver cancer for both sexes and in women gallbladder and oral cancer [13].

Cancer studies which explicitly study the descendants of Pakistani and Bangladeshi immigrants remain scant owing to the young age structure and low number of events. Childhood cancer incidence for children of South Asian and Pakistani descent - who can be assumed to be descendants of migrants - is elevated [39, 40]. In adulthood the risk of infection related cancers, such as stomach and liver which are higher amongst Pakistani and Bangladeshi immigrants, does not affect UK-born descendants to the same extent [13].

Much of this prior research has used broad groupings of South Asian (including Indians) or Asian (including Chinese). At times this can be necessary for quantitative analysis. However, it is important to acknowledge that when combining ethnic and origin groups the heterogeneity in experiences is eroded [9]. The selection and assimilation pathways of Indian and Chinese immigrants has differed substantially from that of Pakistanis and Bangladeshis, with different spatial distributions, socioeconomic outcomes and deviations in health and morbidity [8, 41–45]. Pakistanis and Bangladeshis can justifiably be combined due to similarities in religiosity (majority Muslim) and the shared history prior to the secession of Bangladesh in 1971. But they are geographically separated which can mean different prior exposures and epigenetic development [7, 46]. They also have distinct cultural practices, languages, and values which can influence health behaviours and health literacy [7]. Thus, now that there are substantial populations of Pakistani and Bangladeshi first-generation immigrants it is both theoretically optimal and practically feasible to study them separately.

### Cancer risk factors of Pakistanis and Bangladeshis in England and Wales

When considering the likelihood of cancer incidence and mortality, there are several factors to consider which could differ between the majority population and minority groups.

#### Biological differences

Cancer incidence is lower in Pakistan and Bangladesh [47]. The health transition that immigrants experience should mean the risk of infectious diseases, including infection related cancers, decreases whilst acculturation increases the risk of lifestyle driven diseases [48]. Immigrants are generally positively selected on health characteristics resulting in a healthier immigrant population [18]. They have an epigenetic make-up, shaped by historical factors in the origin country, which persists in the destination [46]. This epigenetic make-up is theoretically inherited by descendants [22]. These differences can be protective characteristics but also negative for example, South Asians have more insulin resistance and higher adiposity than Europeans [49, 50], both cancer risk factors [51].

#### Socioeconomic factors

Socioeconomic disparities are associated with worse cancer survival rates and higher incidence [52–54]. Deprivation is also associated with poorer health and negative health behaviours across the whole population [55]. Discrimination in hiring practices [56], higher unemployment rates [43], and earning gaps [57] are all contributing factors behind higher deprivation rates amongst Pakistanis and Bangladeshis in the UK.

#### Environmental factors

An additional area of disadvantage faced by Pakistani and Bangladeshi communities is residential segregation [44]. Pakistanis and Bangladeshis are generally concentrated in urban areas [58]. This exposes both the immigrant generation and descendants to higher, potentially dangerous, levels of air pollution associated with poorer health and increased neoplasm development [59, 60].

#### Negative health behaviours

Negative habits such as tobacco and alcohol consumption increase the risk of various cancers [61, 62]. Amongst Pakistani and Bangladeshi immigrants smoking rates are very low for women. Pakistani men smoke less than White British men and Bangladeshi men more, although socioeconomic deprivation can explain this difference [63]. Alcohol consumption is substantially lower than in the native population [64] and while alcohol-related mortality has increased, it is still lower than in the White British population [65]. Acculturation to these negative behaviours amongst descendants is observable but they maintain rates lower than the natives [64].

A genetic predisposition to obesity exists for Pakistani and Bangladeshis [50], making diet an important health factor. The traditional diet, high in fat, salt, and oil persists in the immigrant generation [66], contributing to cardiovascular ill-health [67]. The dietary habits of descendants’ shows that these norms continue [64] alongside acculturation to the more negative dietary aspects of high-income countries, such as increased processed food intake [66].

#### Healthcare usage & health beliefs

Survival from cancer can be influenced by healthcare engagement, including participation in screening programs. Among South Asians in the UK, bowel, prostate, and breast screening uptake is lower than the native population. They are even lower amongst Muslim South Asians, a group which includes most Pakistanis and Bangladeshis [68]. Lower knowledge of the existence of these services, which socioeconomic differences alone cannot explain, is considered the reason for this [69, 70]. Additionally, sociocultural beliefs affect the level of fatalism associated with cancer and reduce the perceived importance of screening [71]. Reliance on faith and spiritual practices over modern medicine further limit engagement [72]. Linguistic barriers negatively affect participation in breast, cervical, and colorectal screenings [73, 74]. These inequalities in screening attendance can be an explanation for the slower improvements in breast and prostate cancer survival for Pakistanis and Bangladeshis compared to other groups [6]. These barriers should be less prominent for descendants, who with better language skills and familiarity with the healthcare system should face lower barriers to healthcare access.

### Expectations

Our expectations are that Pakistani and Bangladeshi immigrants will have lower all-cancer incidence relative to the White British group. For subsequent mortality we expect similarly that the protective nature of their epigenetics means lower mortality. Amongst descendants’ we predict incidence to lie between that of first-generation immigrants and the native group, owing to waning maintenance of positive health behaviours. Our expectation is that adjusting for socioeconomic factors will further increase the advantage of lower cancer incidence and mortality for immigrants, at both generation levels, compared to the natives.

## Data and methods

We use the Office for National Statistics-Longitudinal Study (ONS-LS) [75] on a study period which runs from the census of March 1971 until the end of 2016. The ONS-LS is a longitudinal 1% sample of the population of England and Wales. It links census and life event dates such as emigration, re-entry, death, and cancer diagnosis collected from National Health Service (NHS) registrations and de-registrations. An individual becomes part of the ONS-LS if they are born on one of four unspecified birth dates. Cancer information is collected in the ONS-LS via linkage of sample members to the information provided to the English cancer registries and the Welsh Cancer Intelligence and Surveillance Unit [76].

### Sample construction

All members of the ONS-LS born in 1920 or later who participate in at least one census as an adult (aged over 20) are eligible for our study. We further specify our sample using country of birth, parental country of birth and ethnicity. Owing to changes in census methodology over time; parental country of birth is asked only at the 1971 census and ethnic group is available for 1991 onwards and is derived from parental country of birth in 1971.

Natives (also referred to as White British or majority), are defined through having been born in the UK. In addition, if present at the 1971 census, all available parental birth countries must be UK. The United Kingdom in this study includes Channel Islands, Isle of Man, Scotland, and Northern Ireland in addition to England and Wales. Lastly, White or White British (only in later censuses is White British specifically collected) must be the ethnic group.

Immigrants are determined by their country of birth being Pakistan or Bangladesh. The 1971 census combined Pakistan and Bangladesh as a country of origin and ethnic group. Those who appear at multiple censuses are classified using responses from 1981 onwards. Those who only appear in 1971 are reported as “Pakistani/Bangladeshi” thus are not included in the main sample. Self-reported ethnicity must also be Pakistani or Bangladeshi. This prevents White individuals born in Pakistan or Bangladesh from biasing the sample, many of these are children of expatriates born under colonialism in the early 20th century who have different exposures to risk factors and epigenetics, thus different mortality and morbidity profiles [77].

The descendants group combines those with Pakistani and Bangladeshi backgrounds. Combining ensures a sufficient sample size, since the younger age structure of this group means fewer cancer events are observed. We acknowledge that this is a limitation and recommend richer data sources be made available to identify if there are divergences in cancer outcomes between descendants of Pakistani and Bangladeshi immigrants. To be categorised in this group individuals must have the UK as a place of birth and their ethnic group be Pakistani or Bangladeshi. We call this group descendants but most are likely to be second-generation due to the historical migration patterns of Pakistani and Bangladeshis [8], and our requirement of being over age 20.

Census responses to country of birth and ethnic group questions are not necessarily fixed over time [78]. We apply a threshold which requires more than half of available responses to match the criteria for inclusion [12]. For country of birth, in cases when it is exactly half, we adopt the earliest reported. For ethnicity, we are strict with the need for more than half.

**Fig. 1:**
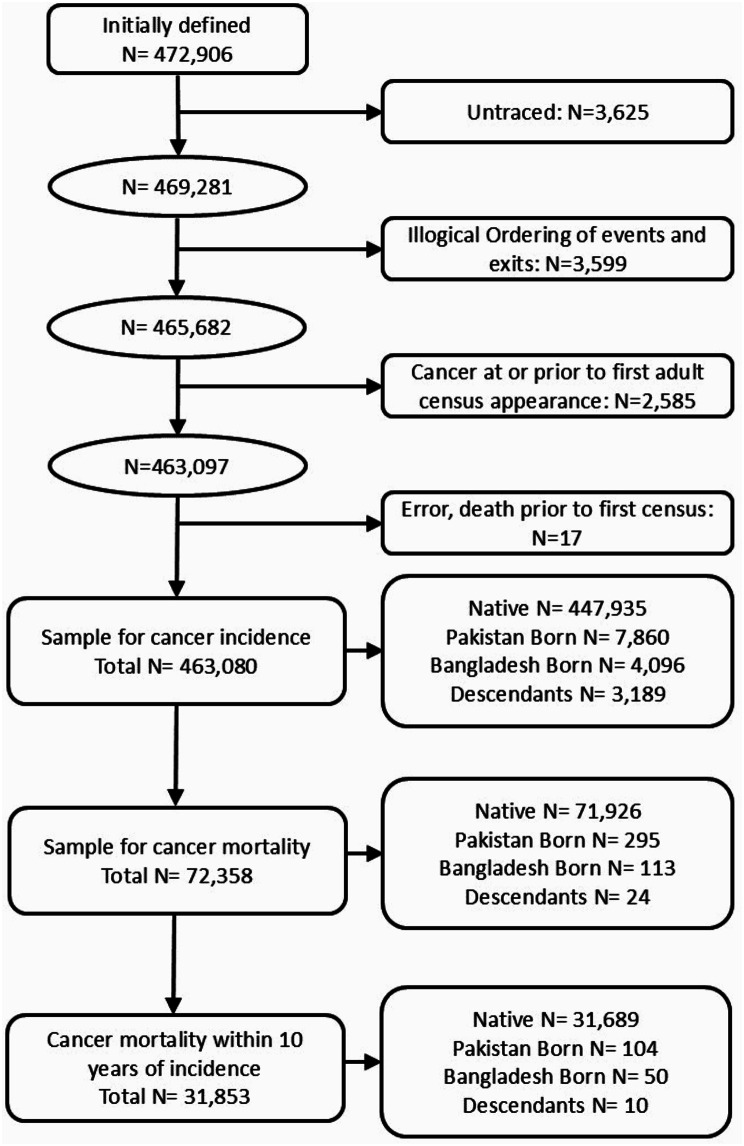
Exclusion criteria and numbers excluded. Note: Initially defined are individuals born 1920–1991, who are present at one census from 1971 to 2011, and match the origins under study. Source: ONS-LS

Initially, 472,906 eligible members met the above requirements. Further exclusions were made on four criteria. First, being untraced (*N* = 3,625), meaning no linkage with the national health service record meaning any cancer event cannot be linked to their census records. Second, those with an illogical ordering of entries and exits (*N* = 3,599). Third, those who had cancer diagnosis prior to their first adult census were excluded (*N* = 2,585). Last, a small number of cases were removed due to erroneous death dates which precede a first census appearance (*N* = 17). Figure 1 details the exclusions to reach a final sample size of 463,080 and further shows the number of events that lead to being in the second analysis studying the mortality risk.

### Outcome measure

We study two events in two different analyses. The first event of interest is a first cancer incidence. Squamous and basal cell carcinomas are reported in the ONS-LS, but these are not included as they are rarely a primary cause of death. Our second analysis investigates subsequent cancer mortality, we use the death dates from the ONS-LS and determine cause of death using International Classifications of Diseases (ICD) codes. The ONS-LS exists over three revisions of ICD codes, 1971 to 1981 is ICD-8, 1981–1999 is ICD-9 and from 2000 onwards has been ICD-10. We harmonise these ICD codes across the sample to dichotomise primary cause of death into either cancer or another cause.

### Covariates

We incorporate a time-fixed covariate for sex and a time-varying covariate related to the time-period of the census. Moreover, further time-varying covariates are included which are assumed to be fixed until the next census. These covariates measure socioeconomic success and demographic behaviours which have been observed as associated with cancer incidence and survival [52–54, 79, 80].

We include location at the time of census, London, Rest of England, or Wales. This control accounts for the devolution of healthcare in Wales. Education is included as a binary measure of degree level or not; this dichotomy was selected to create comparable categories across censuses, which have different categorisation due to changes in education policy. Social class is included as an indicator of socioeconomic status, associated with health inequalities including higher cancer incidence and worse survival [52, 79, 80]. Social class is measured as: technical and managerial, skilled, armed forces, and unskilled. Marital status is also included as better health outcomes amongst married people have been found, however cause of death specific research is less clear [81]. We also include tenure; homeowner (both with and without mortgage), renter, and other, which is typically a ‘group home’ or institutionalisation.

We retain missing categories for social class, tenure, marital status, and location. Missing arises when sample members miss a census through non-completion or non-residence. We impute covariates based on answers from other censuses where it is logical. For example, degree level education is projected forwards and being ‘single never married’ is projected backwards. Table 1 shows the total person-years and events for each covariate.

**Table 1:** Number of events and total exposure time in 1000 person-years for each covariate

	Panel A: Cancer Incidence		Panel B: Death after incidence	
Covariate	Exposure time in 1000 person-years (%)	Events (%)	Exposure time in 1000 person-years (%)	Events (%)
Total	11,092	72,358	331.4	31,853
**Immigrant Background**				
Natives	10885.5 (98.1)	71,926 (99.4)	329.7 (99.5)	31,689 (99.5)
Pakistani-born	125.7 (1.1)	295 (0.4)	1.2 (0.4)	104 (0.3)
Bangladeshi-born	53.9 (0.5)	113 (0.2)	0.4 (0.1)	50 (0.2)
Descendants	26.9 (0.2)	24 (0.0)	0.1 (0.0)	10 (0.0)
**Sex**				
Male	5481.7 (49.4)	33,945 (46.9)	132.2 (39.9)	17,021 (53.4)
Female	5610.3 (50.6)	38,413 (53.1)	199.2 (60.1)	14,832 (46.6)
**Age Band**				
20–25	367.6 (3.3)	411 (0.6)	0.5 (0.2)	10 (0.0)
25–30	988.3 (8.9)	1849 (2.6)	4.8 (1.4)	72 (0.2)
30–35	1272.6 (11.5)	2343 (3.2)	12.4 (3.7)	196 (0.6)
35–40	1301.9 (11.7)	2477 (3.4)	17.3 (5.2)	332 (1.0)
40–45	1307.5 (11.8)	3023 (4.2)	18.6 (5.6)	745 (2.3)
45–50	1278.5 (11.5)	4304 (5.9)	21.5 (6.5)	1288 (4.0)
50–55	1204.4 (10.9)	5980 (8.3)	26.9 (8.1)	2268 (7.1)
55–60	1014.6 (9.1)	7552 (10.4)	32.9 (9.9)	3262 (10.2)
60–65	822.6 (7.4)	9373 (13.0)	39.5 (11.9)	4251 (13.3)
65–70	625 (5.6)	10,363 (14.3)	43.7 (13.2)	5022 (15.8)
70–75	429.2 (3.9)	9656 (13.3)	41.8 (12.6)	4911 (15.4)
75–80	270.2 (2.4)	7819 (10.8)	35.4 (10.7)	4502 (14.1)
80–85	142.8 (1.3)	4751 (6.6)	23.5 (7.1)	3082 (9.7)
85+	66.8 (0.6)	2457 (3.4)	12.5 (3.8)	1912 (6.0)
**Census Period**				
1971–1981	2057.4 (15.8)	3364 (4.6)	8.9 (2.7)	1468 (4.6)
1981–1991	2715.7 (20.8)	8470 (11.7)	30.6 (9.2)	4048 (12.7)
1991–2001	3259.8 (25.0)	18,462 (25.5)	75.7 (22.8)	8472 (26.6)
2001–2011	3304.1 (25.3)	27,411 (37.9)	134.5 (40.6)	11,670 (36.6)
2011–2016	1712.4 (13.1)	14,651 (20.2)	81.7 (24.7)	6195 (19.4)
**Education**				
Degree	1374.7 (12.4)	8500 (11.7)	46.8 (14.1)	2692 (8.5)
No Degree	9717.3 (87.6)	63,858 (88.3)	284.6 (85.9)	29,161 (91.5)
**Social Class**				
Professional, technical, and managerial	2783.1 (25.1)	16,443 (22.7)	83.8 (25.3)	5686 (17.3)
Skilled	5623.4 (50.7)	34,323 (47.4)	159.4 (48.1)	14,315 (43.6)
Unskilled	528.7 (4.8)	3657 (5.1)	15.9 (4.8)	2748 (8.4)
Armed Forces	29.9 (0.3)	76 (0.1)	0.4 (0.1)	33 (0.1)
Missing	2126.9 (19.2)	17,859 (24.7)	71.9 (21.7)	10,071 (30.7)
**Location**				
London	1089.5 (9.8)	5534 (7.6)	22.3 (6.7)	2621 (8.2)
Rest of England	9011.3 (81.2)	58,986 (81.5)	276 (83.3)	25,132 (78.9)
Wales	649.7 (5.9)	4438 (6.1)	20.4 (6.2)	1941 (6.1)
Unknown/Missing	341.5 (3.1)	3400 (4.7)	12.6 (3.8)	2159 (6.8)
**Marital Status**				
Never Married	2425.9 (21.9)	8214 (11.4)	34.7 (10.5)	2616 (8.0)
Married	7175.9 (64.7)	46,925 (64.9)	217.4 (65.6)	20,127 (61.9)
Widowed	408.4 (3.7)	7792 (10.8)	34.8 (10.5)	4667 (14.3)
Divorced	798.1 (7.2)	6455 (8.9)	33.3 (10.0)	2578 (7.9)
Missing	283.7 (2.6)	2972 (4.1)	11.2 (3.4)	2553 (7.8)
**Tenure**				
Owner Occupied	7355.7 (66.3)	48,797 (67.4)	238.9 (72.1)	19,607 (61.6)
Renter	3196.4 (28.8)	18,830 (26.0)	74 (22.3)	9244 (29.0)
Other	170.9 (1.5)	930 (1.3)	4.2 (1.3)	617 (1.9)
Missing	368.9 (3.3)	3801 (5.3)	14.4 (4.3)	2385 (7.5)

Source: Authors’ calculations using ONS-LS

### Method and models

The analyses were conducted in Stata version 17. We apply survival analysis and Cox proportional hazard models to; first, estimate the risk of cancer incidence and second, estimate the risk of cancer mortality after incidence. Our baseline time is measured as months since turning age 20. All individuals become at risk at the date of their first census appearance when aged over 20. Information on immigration before a first census is available, linked through the date of registration with the NHS. However, using this date would create bias since those who do register could be in worse health as they are seeking medical treatment. Moreover, since the covariables are only collected at censuses, including immigrants at their arrival date would result in more missing amongst covariates.

Individuals are censored at death and emigration. Individuals with no information relating to death or emigration following their final census appearance are deemed ‘lost to follow up’ (LTFU). These individuals are apportioned four years of exposure time following their final census appearance which is deemed the optimal amount of time based on the exit dates available in the sample [82]. The exception to this is after 2011 when we assume survival to the end of the study period, which is the end of 2016. We allow for entry and exit to the sample based on the emigration dates and re-entry dates which are linked to NHS health records. We use the mid-point of dates when there is missing information, for example estimating the exit date when we have two re-entry dates and no exit date between them, or two exit dates but no re-entry date.

For incidence we have a base model (Model 1) which only controls for sex and the time-period. Model 2 introduces controls for location, social class and education. The full model (Model 3) includes tenure and marital status. The co-variates added in Model 2 attempt to capture individual level characteristics and Model 3 are related to household level characteristics, and follows the stepwise approach of migrant mortality work that has used the ONS-LS [12].

To study the risk of mortality after incidence the sample is restricted to those who experience a cancer incidence during the study period, *N* = 72,358. These individuals are followed for a maximum of 10 years from that date, with the event of interest being a death where the primary cause is cancer. Censoring occurs at emigration, LTFU, death from another cause, and the end of 2016. 4,424 individuals are reported as dying in the same month of their cancer incidence. In this case we allocate half a month of exposure time. A sensitivity analysis where 0.03 months (approx. one day) was allocated did not impact the results.

The analysis of mortality uses a baseline of time since diagnosis, instead of age, thus we include five-year age bands in the base model (Model 1). Moreover, due to different prognoses of different cancers we introduce a co-variate for the site the cancer is diagnosed at (Model 2). Further models follow the stepwise approach of the analysis of incidence; Model 3 adds location, social class, and education. Model 4 includes tenure and marital status also.

## Results

### Risk of a cancer incidence

**Fig. 2:**
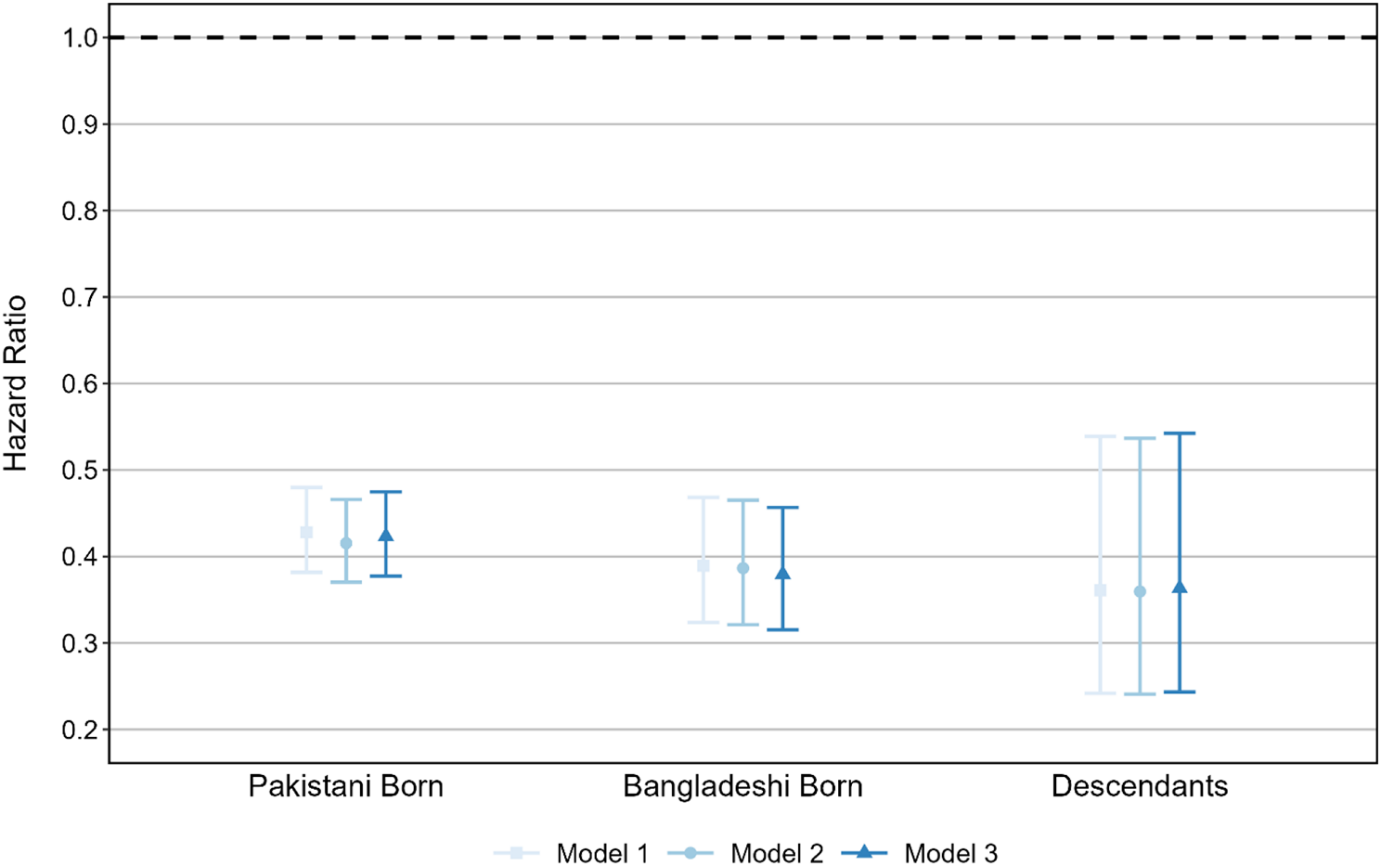
Hazard ratios of first adult cancer incidence. Note: Reference category is White British. 95% Confidence intervals shown. Source: Authors’ calculations using ONS-LS

Results are presented in Fig. 2 showing hazard ratios (HR) for the risk of cancer incidence for each background and each model. These HRs are in reference to the White British population. Incidence of cancer amongst Pakistani-born, Bangladeshi-born, and their descendants is substantially lower than amongst White British. In Model 1 the HR of cancer onset for immigrants born in Pakistan is 0.43 (95% confidence interval (CI): 0.38–0.48) and for those born in Bangladesh 0.39 (CI: 0.32–0.47). Amongst descendants the ratio is 0.36 (CI: 0.24–0.54).

The introduction of covariates does little to change the magnitude of the association, with much lower rates remaining for across all three models. In the fully adjusted model, the Pakistan-born experience HR of 0.42 (CI: 0.38–0.47). Amongst the Bangladesh-born it is even smaller at 0.38 (CI: 0.32–0.46) and descendants have HR of 0.36 (CI: 0.24–0.54) compared to natives.

The HRs of the covariates (see Table 2) follow expected there is increased risk of incidence in later time-periods. This is to be expected given better cancer detection through screening programs and the ageing of the sample. There are clear socioeconomic gradients with reduced risk of cancer incidence amongst those; in higher occupational classes, with degree level education and, living in owner occupied properties. Rates by marital status show limited differences, divorced and separated individuals have elevated risk of cancer incidence, whilst widowhood is associated with lower risk. No difference is observed between those never married and married.

**Table 2:** Cox proportional hazard model: first cancer incidence in adulthood

	Model 1		Model 2		Model 3	
	HR	95% CI	HR	95% CI	HR	95% CI
Immigrant Background						
Natives	1	N/A	1	N/A	1	N/A
Pakistani-born	0.43	0.38–0.48	0.42	0.37–0.47	0.42	0.38–0.47
Bangladeshi-born	0.39	0.32–0.47	0.39	0.32–0.47	0.38	0.32–0.46
Descendants	0.36	0.24–0.54	0.36	0.24–0.54	0.36	0.24–0.54
Sex						
Male	1	N/A	1	N/A	1	N/A
Female	1.01	0.99–1.02	1.00	0.98–1.01	1.01	0.99–1.02
Time Period						
1971–1981	1	N/A	1	N/A	1	N/A
1981–1991	1.28	1.23–1.33	1.27	1.22–1.32	1.29	1.23–1.34
1991–2001	1.66	1.60–1.72	1.65	1.59–1.72	1.70	1.64–1.77
2001–2011	1.93	1.86-2.00	1.94	1.87–2.02	2.00	1.93–2.08
After 2011	1.94	1.86–2.02	2.01	1.93–2.09	2.06	1.97–2.14
Location						
London			1	N/A	1	N/A
Rest of England			1.10	1.07–1.13	1.12	1.09–1.15
Wales			1.12	1.07–1.16	1.14	1.09–1.18
Missing			1.29	1.23–1.35	1.38	1.21–1.58
Social Class						
Managerial, Technical and Professional			1	N/A	1	N/A
Skilled			1.04	1.02–1.06	1.03	1.00-1.05
Unskilled			1.00	0.96–1.04	0.96	0.92–0.99
Armed Forces			0.77	0.62–0.97	0.77	0.62–0.97
Missing/Other			1.09	1.06–1.11	1.05	1.03–1.08
Education						
No Degree			1	N/A	1	N/A
Has Degree			0.94	0.91–0.96	0.95	0.92–0.97
Marital Status						
Never married					1	N/A
Married					1.02	0.99–1.04
Widowed					0.93	0.90–0.96
Divorced/Separated					1.08	1.04–1.11
Missing					0.89	0.81–0.99
Tenure						
Owner Occupied					1	N/A
Rented					1.17	1.15–1.19
Other					0.88	0.82–0.94
Missing					1.10	1.00-1.21

Source: Authors’ calculations using ONS-LS

Survival bias from the censoring of individuals at mortality prior to cancer incidence could influence these findings. In theory, these deaths are found in the unhealthiest individuals who would be more likely to develop cancer later in the life course. There is evidence of elevated risk of cardiovascular disease amongst Pakistanis and Bangladeshis [67] alongside more deaths at younger ages amongst descendants [83]. Therefore, it is feasible that those under study at older ages, when cancer is more prevalent, are healthier overall. To test this survival bias we ran a model (specified as Model 3) to estimate the relative risk of mortality prior to cancer incidence. This found that the risk of mortality prior to cancer incidence is significantly lower for those born in Pakistan and Bangladesh. For descendants there was no significant difference. Thus, if there is a bias due to the censoring of unhealthy individuals prior to cancer incidence it is more prevalent in the native population and thus the true HRs would be even smaller than the results shown.

### Risk of mortality after cancer incidence

**Fig. 3:**
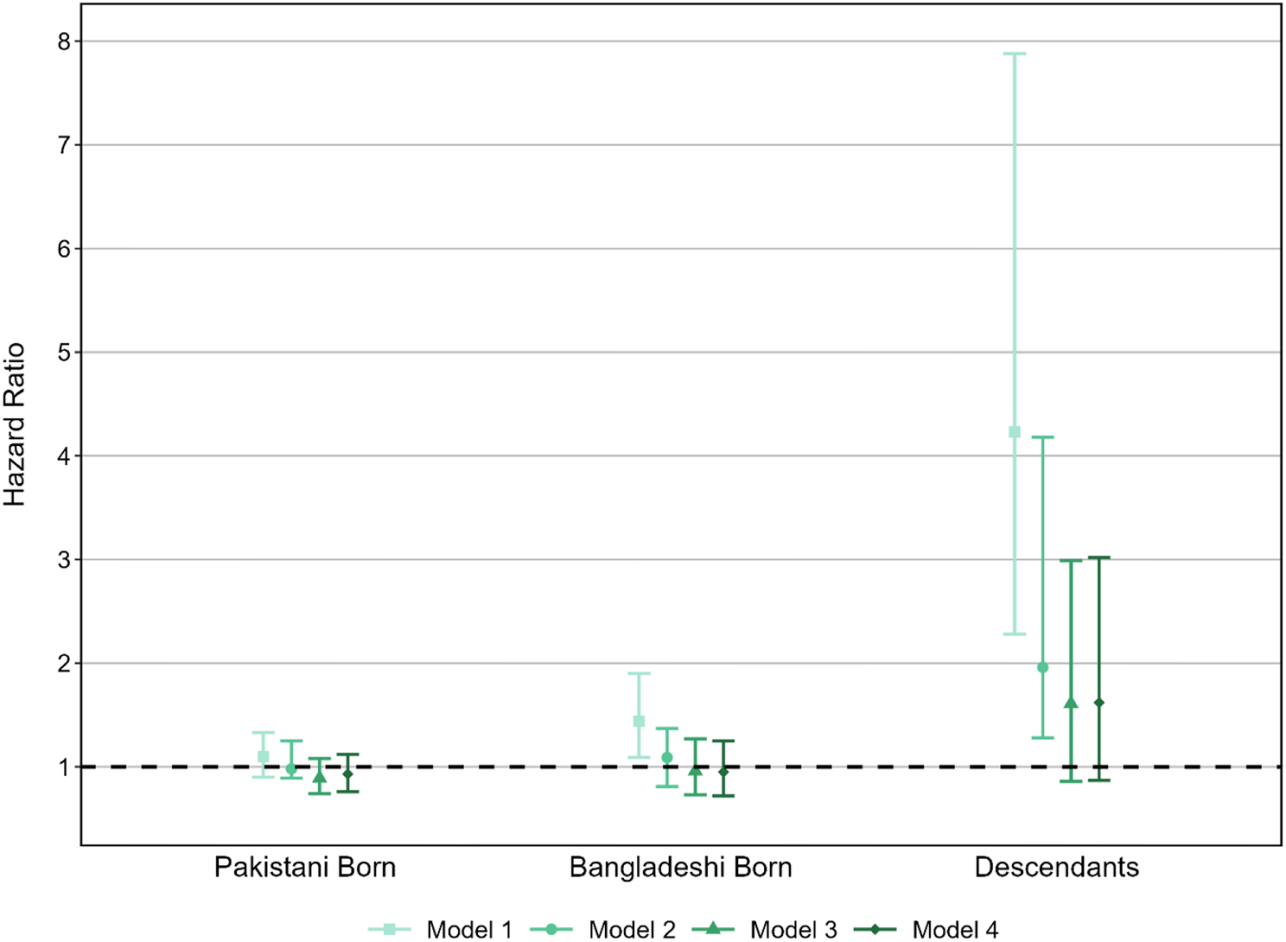
Hazard Ratios of cancer death following incidence. Note: Reference category is White British. 95% Confidence intervals shown. Source: Authors’ calculations using ONS-LS

Main results of the second analysis, on cancer mortality in the ten years following incidence, are in Fig. 3. In Model 1 which controls only for sex, five-year age band, and time-period we find no significant difference for Pakistani-born immigrants (HR 1.10, CI: 0.90–1.33). For immigrants born in Bangladesh the HR is 1.44 (CI: 1.09–1.90). Descendants have a substantially elevated HR of 4.23, but the confidence interval is wide as there are so few incidences in the first place (CI: 2.28–7.88).

The addition of a variable for the site of the cancer (Model 2) reduces the HR for Pakistani-born individuals to 0.98, but it remains non-significant (CI: 0.89–1.25). The introduction of this control explains the relative mortality difference for Bangladeshi immigrants with HR 1.09 (CI: 0.81–1.37). For the descendants, the control also substantially reduces the HR down to 1.96 (CI: 1.28–4.18).

Model 3 and Model 4 show very similar results, therefore we only mention the results of the fully adjusted model (Model 4). We find no significant (at 95%) differences between any of the groups under study. The Pakistani-born immigrants have a HR of 0.93 (CI: 0.76–1.12). For those born in Bangladesh it is similar at 0.95 (CI: 0.72–1.25). Amongst descendants mortality compared to natives remains elevated with HR of 1.62 (CI: 0.87–3.02) – this result is significantly different from the natives at 90% confidence.

Full results can be found in Table 3. Patterns for covariates are in line with expectations, the risk of death after incidence increases with age and deceases across time-periods, a symptom of the better treatment which has increased cancer survival. Gradients by social class are apparent, skilled and unskilled both have higher relative risk of mortality after onset compared to those defined in the highest social class. Moreover, those who obtain degree level education have a lower risk of cancer death following incidence. Differences by marital status show that being ever married, even if now divorced or widowed, is associated with lower risk of cancer mortality following incidence compared to those never married.

**Table 3:** Cox proportional hazard model: cancer mortality following incidence

	Model 1		Model 2		Model 3		Model 4	
	HR	95% CI	HR	95% CI	HR	95% CI	HR	95% CI
Immigrant Background								
Natives	1	N/A	1	N/A	1	N/A	1	N/A
Pakistani-born	1.10	0.90–1.33	0.98	0.89–1.25	0.89	0.74–1.08	0.93	0.76–1.12
Bangladeshi-born	1.44	1.09–1.90	1.09	0.81–1.37	0.96	0.73–1.27	0.95	0.72–1.25
Descendants	4.23	2.28–7.88	1.96	1.28–4.18	1.61	0.86–2.99	1.62	0.87–3.02
Age Band								
20–25	0.14	0.07–0.26	0.24	0.13–0.44	0.23	0.12–0.42	0.20	0.11–0.38
25–30	0.15	0.11–0.18	0.24	0.19–0.30	0.24	0.19–0.30	0.21	0.17–0.27
30–35	0.21	0.18–0.25	0.32	0.27–0.37	0.32	0.27–0.37	0.30	0.26–0.35
35–40	0.30	0.26–0.33	0.39	0.35–0.44	0.39	0.35–0.44	0.38	0.34–0.43
40–45	0.55	0.51–0.60	0.67	0.61–0.73	0.66	0.61–0.72	0.66	0.60–0.71
45–50	0.75	0.70–0.80	0.80	0.75–0.86	0.80	0.75–0.86	0.80	0.75–0.86
50–55	1	N/A	1	N/A	1	N/A	1	N/A
55–60	1.23	1.17–1.30	1.13	1.07–1.19	1.12	1.06–1.19	1.12	1.06–1.18
60–65	1.41	1.34–1.49	1.27	1.20–1.33	1.25	1.18–1.31	1.25	1.18–1.31
65–70	1.62	1.53–1.70	1.44	1.37–1.52	1.40	1.33–1.47	1.40	1.33–1.47
70–75	1.78	1.69–1.88	1.61	1.53–1.70	1.54	1.46–1.63	1.54	1.46–1.62
75–80	2.13	2.02–2.25	2.01	1.91–2.12	1.88	1.78–1.99	1.87	1.77–1.97
80–85	2.56	2.42–2.71	2.46	2.32–2.61	2.19	2.06–2.32	2.16	2.03–2.30
85+	3.26	3.06–3.48	3.21	3.01–3.43	2.83	2.64–3.02	2.74	2.56–2.94
Sex								
Male	1	N/A	1	N/A	1	N/A	1	N/A
Female	0.77	0.75–0.79	0.87	0.85–0.89	0.83	0.81–0.85	0.83	0.80–0.85
Time Period								
1971–1981	1	N/A	1	N/A	1	N/A	1	N/A
1981–1991	0.70	0.65–0.74	0.77	0.72–0.82	0.76	0.72–0.81	0.77	0.72–0.82
1991–2001	0.49	0.46–0.52	0.62	0.58–0.66	0.62	0.59–0.66	0.63	0.60–0.67
2001–2011	0.33	0.31–0.35	0.45	0.43–0.48	0.47	0.44–0.49	0.48	0.45–0.51
After 2011	0.28	0.26–0.30	0.38	0.35–0.40	0.42	0.39–0.44	0.42	0.39–0.45
Cancer Type								
Colorectal			1	N/A	1	N/A	1	N/A
Bronchus/Lung			3.64	3.49–3.79	3.55	3.40–3.70	3.48	3.33–3.63
Prostate			0.48	0.45–0.51	0.48	0.46–0.51	0.49	0.46–0.52
Kidney			1.27	1.17–1.38	1.27	1.17–1.39	1.26	1.16–1.37
Bladder			0.69	0.64–0.74	0.69	0.64–0.74	0.68	0.64–0.74
Stomach			2.71	2.54–2.89	2.64	2.48–2.82	2.61	2.45–2.79
Non-Hodgkin’s lymphoma			1.00	0.92–1.07	1.00	0.93–1.08	1.00	0.93–1.08
Melanoma/Skin			0.44	0.40–0.49	0.45	0.41–0.51	0.46	0.41–0.51
Pancreatic			5.15	4.81–5.50	5.14	4.81–5.49	5.14	4.81–5.49
Leukaemia			1.32	1.21–1.43	1.32	1.22–1.43	1.32	1.22–1.43
Oesophageal			2.95	2.75–3.16	2.92	2.72–3.13	2.87	2.68–3.08
Oral			0.94	0.86–1.03	0.92	0.84–1.01	0.91	0.83-1.00
Brain			4.25	3.94–4.59	4.31	4.00-4.66	4.33	4.01–4.67
Myeloma			1.52	1.37–1.67	1.52	1.37–1.67	1.51	1.37–1.67
Liver			4.29	3.88–4.75	4.24	3.83–4.70	4.25	3.84–4.70
Thyroid			0.50	0.40–0.63	0.51	0.41–0.64	0.52	0.41–0.65
Breast			0.59	0.56–0.62	0.59	0.56–0.62	0.60	0.57–0.63
Uterine			0.49	0.44–0.54	0.49	0.44–0.54	0.49	0.44–0.54
Ovary			1.68	1.56–1.81	1.68	1.56–1.81	1.69	1.57–1.82
Cervical			0.90	0.81-1.00	0.88	0.79–0.97	0.87	0.78–0.96
Other malignant neoplasm			0.43	0.41–0.45	0.43	0.42–0.45	0.43	0.42–0.45
Location								
London					1	N/A	1	N/A
Rest of England					0.92	0.88–0.96	0.94	0.90–0.98
Wales					0.93	0.88–0.99	0.96	0.90–1.02
Missing					1.15	1.09–1.23	1.15	0.96–1.38
Social Class								
Managerial, technical, and professional					1	N/A	1	N/A
Skilled					1.14	1.10–1.18	1.12	1.08–1.16
Unskilled					1.24	1.18–1.31	1.18	1.12–1.25
Armed Forces					1.04	0.74–1.46	1.02	0.72–1.44
Missing/Other					1.33	1.28–1.38	1.27	1.22–1.32
Education								
No Degree					1	N/A	1	N/A
Has Degree					0.88	0.85–0.92	0.90	0.86–0.94
Marital Status								
Never married							1	N/A
Married							0.88	0.84–0.92
Widowed							0.97	0.92–1.02
Divorced/Separated							0.90	0.85–0.96
Missing							0.77	0.68–0.87
Tenure								
Owner Occupied							1	N/A
Rented							1.15	1.16–1.22
Other							1.49	1.62–1.84
Missing							1.25	1.17–1.45

Source: Authors’ calculations using ONS-LS

### Sensitivity analyses

We ran several sensitivity analyses to investigate different sample specifications, particularly relating to using different inclusion criteria given potential changes over time in self-reported ethnicity [78]. Descriptions, sample sizes and the HR for a first cancer incidence are in ‘Additional Tables 1 & 2, Additional Results’. None of these specifications alter interpretation of the results. Further since the use of ‘missing’ as a category generates scepticism in health research [84], we repeated the analysis using only complete cases, the results hold with only small changes to the magnitude, see ‘Additional Table 3, Additional Results’.

We considered alternative ways of capturing socioeconomic status by using economic position as a covariate instead of, and as well as, social class, the differences are minimal, see ‘Additional Table 4, Additional Results’. Due to the small number of events and data restrictions we maximise sample size and statistical power by using a non-stratified sample, with sex as a covariate. However, socioeconomic determinants of health, and therefore susceptibility to cancer, differ by sex [85]. Sex stratified models are in ‘Additional Tables 5 & 6, Additional Results’. The results are generally stable, but for male descendants there is no longer a significant difference in cancer incidence compared to the natives. The risk of mortality for descendants also finds that men have a higher relative risk compared to women. For Bangladeshi immigrants, the opposite is found, women born in Bangladesh show higher relative risk of cancer mortality after incidence than men born in Bangladesh.

Lastly, we considered the age structure. The foreign-born and natives have similar time at risk within each age band, however little observation time of descendants is after age 50. To compensate for this difference, we repeated both sets of analyses censoring all observations at age 50. The results of the fully adjusted models using this specification are in ‘Additional Tables 7 & 8, Additional Results’, they are consistent with the main findings.

## Discussion

This study supports previous findings of low cancer incidence and mortality in Pakistani and Bangladeshi-born individuals [11, 12]. Our approach looks at both incidence and subsequent mortality using one dataset; in doing so we can add certainty that low cancer mortality is driven by low incidence not by better survival. We find evidence which suggests that low incidence persists between generations. In the ten years following diagnosis there is little evidence to suggest that cancer mortality differs between any group. However, there is some weak evidence that mortality after onset is elevated for the descendants of Pakistani and Bangladeshi immigrants. This is based on a small sample so caution should be taken when interpreting this group.

We found substantial differences in cancer incidence between the immigrant groups and the native population, confirming our expectations. Building on previous research which has identified lower incidence amongst (South) Asians as a broad group and lower incidence for specific cancer sites [4, 15–17]. Our analysis finds that this advantage persists to UK-born descendants, this was somewhat expected but not to this magnitude. We speculate that these findings are reflective of both environmental factors related to the lower burden of cancer in the origin [47]. Alongside the maintenance of healthy behaviours [20], particularly low alcohol and tobacco usage [63, 64]. Low cancer incidence for descendants can be a combination of inheritance of the positive selection from their immigrant parents and a continuation of these healthy behaviours. This continuation could be indicative of the low socialisation with the native population, which has led to entrenched behavioural norms and avoided ‘unhealthy assimilation’ [35]. Our findings are robust to survival bias owing to excess rates of cardiovascular disease mortality amongst Pakistani and Bangladeshis [12, 67, 86].

We find that this advantage is only present for cancer incidence, against our expectations. There are no significant differences in cancer mortality after onset between groups. Therefore, any epigenetic advantage or health protective behaviours are influential to onset rather than survival. Alternatively, the universal health care system of England and Wales could be acting as an equaliser across society [87]. Universal health care incorporates screening programs; previous research has found that these are less utilised by Pakistani and Bangladeshis [68, 88]. Whilst this might be due to a, potentially justified, belief that cancer is less prevalent in their communities [71] it may lead to late detection and therefore worse prognoses, undoing potential genetic advantage.

The use of socioeconomic variables does little to change the magnitude of the results for cancer incidence for any of the observed groups. Given the relatively worse socioeconomic outcomes of Pakistani and Bangladeshis [56] this was surprising. When analysing cancer mortality after incidence, the inclusion of socioeconomic controls does change the hazard ratios of Pakistani and Bangladeshi immigrants to suggest lower mortality than natives - but it remains non-significant. For descendants, the inclusion of these covariates does explain the elevated mortality [83], indicating that the negative socioeconomic experiences of minority ethnic Pakistanis and Bangladeshis is negatively impacting health.

The mortality analysis has a relatively small number of cancer onsets, therefore we await a time when enough descendants have reached peak cancer and mortality ages, to see if accumulated disadvantage across the life course has negatively affected their longevity. Amongst descendants, the covariate which had the largest influence on the results was the site of the cancer. Due to the low numbers of incidence, there is limited scope to discuss the types of cancer affecting the groups, which future research with better administrative data should attempt to rectify. We do find that descendants are being inflicted with cancers in the early life course that have worse prognoses, compared to older sample members. This is why we included a sensitivity analysis that censored individuals at age 50, this still found weak (at 90%) significance that the cancer mortality for descendants after onset compared to native reference group is elevated, so we can tentatively say that there is evidence that descendants are getting more aggressive cancers in the early life.

Other limitations of this study do exist. We use a rich source of representative administrative data, but census questions do not pertain to behaviours. Therefore, we can only speculate on the persistence of health behaviours as a reason for low cancer incidence amongst the Pakistani and Bangladeshi group. Moreover, due to the decennial nature of the census our socioeconomic variables are presumed fixed for ten years until the next census, thus the exposure time for each covariate is not totally accurate, given the limited effect of socioeconomic variables on the main results this concern is minimal though.

## Conclusion

What our study contributes is a clear overview of the all-cancer incidence and subsequent mortality differences between natives, Pakistanis, Bangladeshis, and their descendants. We have done so in a way that respects the distinction of Pakistanis and Bangladeshis, whilst our results can justify their combination, we maintain that it is always preferrable to separate ethnic and migrant origins if possible. Unfortunately, due to low event counts investigating the descendants of Pakistanis and Bangladeshis separately was not possible, and with evidence of divergence between these groups in other life domains [42] this should re-visited in the future.

To our knowledge, this is the first research using individual-level data that takes a generational approach. In doing so we find the persistence of low cancer incidence between generations, which can be an indicator of a lack of assimilation and acculturation. Overall, our findings can be used to confirm that previous findings of low all-cancer mortality in Pakistani and Bangladeshi immigrants is due to lower incidence and not better survival.

## Electronic supplementary material

Below is the link to the electronic supplementary material.

Supplementary Material 1: Additional Results: This file contains 8 Tables across five sheets. These tables contain results and description related to sensitivity analyses that are outlined primarily in Sect. 3.3

## Data Availability

The datasets supporting the conclusions of this article are available from Office for National Statistics – Longitudinal Study. Restrictions apply to the availability of these data, which were used under license for the current study, and are not publicly available.

## Abbreviations

BB: Bangladeshi-born
CI: Confidence Interval
HR: Hazard Ratio
ICD: International Classifications of Diseases
LTFU: Lost to follow up
ONS-LS: Office for National Statistics – Longitudinal Study
PB: Pakistani-born
UK: United Kingdom
WB: White British
