# Correction: All-cancer incidence and mortality in Pakistanis, Bangladeshis, and their descendants in England and Wales

**DOI:** 10.1186/s12889-024-20967-y

**Published:** 2024-12-18

**Authors:** Joseph Harrison, Frank Sullivan, Katherine Keenan, Hill Kulu

**Affiliations:** 1 School of Geography and Sustainable Development, University of St Andrews, Irvine Building, North Street, St Andrews, KY16 9AL UK; 2 School of Medicine, University of St Andrews, St Andrews, UK

**Correction: BMC Public Health (2024) 24:3352**

10.1186/s12889-024-20813-1

The original publication of this article contained an incorrect funding section. The incorrect and correct information is shown in this correction article, the original article has been updated.

Incorrect

The study is funded by the University of St Andrews. 

Correct

The study is funded by the St Leonard’s Postgraduate College, University of St Andrews. Additionlly, this paper is part of the MigrantLife project that has received funding from the European Research Council (ERC) under the European Union’s Horizon 2020 research and innovation programme (Grant agreement No. 834103).

